# Temperature and work: Time allocated to work under varying climate and labor market conditions

**DOI:** 10.1371/journal.pone.0254224

**Published:** 2021-08-25

**Authors:** Matthew Neidell, Joshua Graff Zivin, Megan Sheahan, Jacqueline Willwerth, Charles Fant, Marcus Sarofim, Jeremy Martinich

**Affiliations:** 1 Department of Health Policy and Management, Columbia University, New York, New York, United States of America; 2 Department of Economics, University of California at San Diego, La Jolla, California, United States of America; 3 Industrial Economics, Cambridge, Massachusetts, United States of America; 4 U.S. Environmental Protection Agency, Washington, District of Columbia, United States of America; TED University, TURKEY

## Abstract

Workers in climate exposed industries such as agriculture, construction, and manufacturing face increased health risks of working on high temperature days and may make decisions to reduce work on high-heat days to mitigate this risk. Utilizing the American Time Use Survey (ATUS) for the period 2003 through 2018 and historical weather data, we model the relationship between daily temperature and time allocation, focusing on hours worked by high-risk laborers. The results indicate that labor allocation decisions are context specific and likely driven by supply-side factors. We do not find a significant relationship between temperature and hours worked during the Great Recession (2008–2014), perhaps due to high competition for employment, however during periods of economic growth (2003–2007, 2015–2018) we find a significant reduction in hours worked on high-heat days. During periods of economic growth, for every degree above 90 on a particular day, the average high-risk worker reduces their time devoted to work by about 2.6 minutes relative to a 90-degree day. This effect is expected to intensify in the future as temperatures rise. Applying the modeled relationships to climate projections through the end of century, we find that annual lost wages resulting from decreased time spent working on days over 90 degrees across the United States range from $36.7 to $80.0 billion in 2090 under intermediate and high emission futures, respectively.

## Introduction

Climate change is expected to warm the earth considerably in the coming decades. These warmer temperatures are projected to increase human mortality and morbidity through a range of direct and indirect channels. There is also growing evidence that extreme temperatures can affect the ability to work through its impacts on endurance and fatigue [1–7] particularly for workers in climate exposed industries, such as agriculture, construction, and manufacturing.

In this paper, we estimate the impacts of climate on the allocation of time to labor, extending the pioneering work of Graff-Zivin and Neidell (hereafter, GZN) [8], who found that extreme heat led to significant declines in labor hours worked in climate-exposed industries, in two important ways. First, we explore whether the relationship found in GZN holds over a longer time period. GZN was based on data from four years, 2003–2006. Since that publication, an additional 12 years of data have become available.

Second, we exploit the significant changes in economic conditions that occurred during this extended time period to assess how this relationship varies with the business cycle. GZN was conducted solely during a period of economic growth, with ample labor market opportunities that may have made it easier for workers to negotiate a reduction in hours on extremely hot days.

Our expanded dataset not only includes periods of economic growth but also a period of significant economic contraction that likely diminished worker bargaining power, making it harder for workers to demand a reduction in hours when environmental conditions are less hospitable. As such, comparing the results from recessionary and non-recessionary periods can shed light on whether these changes in hours worked are driven by supply-side considerations from workers or demand-side factors from the firm.

Using data on time allocation from the American Time Use Survey (ATUS) linked to daily weather data, we conduct our analysis separately for periods of economic growth (2003–2007, 2015–2018) and the contraction that occurred during the Great Recession (2008–2014), focusing solely on workers in climate-exposed industries. For the periods of economic growth, our results are quite similar to those found in GZN from a more limited time span, providing important evidence on the robustness of the negative relationship between extreme temperature and hours worked in exposed sectors. In contrast, we find that extreme temperatures do not lead to changes in hours worked during the Great Recession, underscoring the importance of worker bargaining power and supply-side concerns in shaping labor responses to extreme temperatures. Applying the results from the non-recession years to the expected periods of economic expansion over the remainder of the 21^st^ century under a range of future climate scenarios, the time allocated to labor could decrease by up to 1.5 percent per worker in highly exposed industries (hereafter, high-risk workers) by 2090, resulting in lost wages totaling $80.0 billion annually across all high-risk workers in the continental United States (2015 dollars).

## Data

### Time use data

To characterize time devoted to work at the individual level, this analysis relies on data from the ATUS for the 16-year period between 2003 and 2018. ATUS is a nationally representative cross-sectional survey describing how Americans over age 15 spend their time. Respondents complete a detailed diary of how they allocated the preceding 24 hours by activity, location, and length of time (down to the minute).

This analysis follows the same methodology employed by GZN to identify and aggregate across activities defined as work. We define work as all activities under the “work and work-related activities” major category. In addition to time spent at the workplace, it also includes time devoted to other income-generating activities as well as job searching. We limit our sample to high-risk workers based on their exposure to weather or non-climate-controlled environments. Following the same classification used in GZN, we define these individuals using ATUS industry codes that identify workers in: agriculture, forestry, fishing, hunting (4 percent of high-risk workers); mining (1 percent); construction (26 percent); manufacturing (47 percent); and transport and utilities (22 percent) sectors. Because the diary day could occur on any date, the model controls for day of week, whether the diary date was a holiday, and whether the respondent was employed but absent from work.

### Location and demographic data

ATUS respondents are a random sub-sample of Current Population Survey (CPS) monthly respondents, enabling the ATUS data to link with the CPS data, which contains various demographic variables as well as location information. For this analysis, ATUS responses need location assignment, by county, to map diary entries to weather data. To maintain confidentiality, location information is only released for individuals from areas with over 100,000 residents. County of residence is provided for nearly 40 percent of the respondents targeted by ATUS for the time period of interest in this study. CPS mapping adds county information to an additional six percent of the observations. Thirty-two percent of observations have no county information but identify Census Based Statistical Area (CBSA), New England City and Town Area (NECTA), or Primary Metropolitan Statistical Area (PMSA). We use Census delineation files, which map Census places to counties, and 2010 Census populations to identify and assign the most populous county per statistical area to respondents. Locations are identified for 76 percent of all ATUS respondents (153,366 of 201,151 observations).

The CPS and ATUS together also provide other demographic information for the respondents used as control variables in our analysis. This information includes age, gender, number of children, income, race and ethnicity, education level, and marital status.

### Weather and daylight data

**Fig 1:**
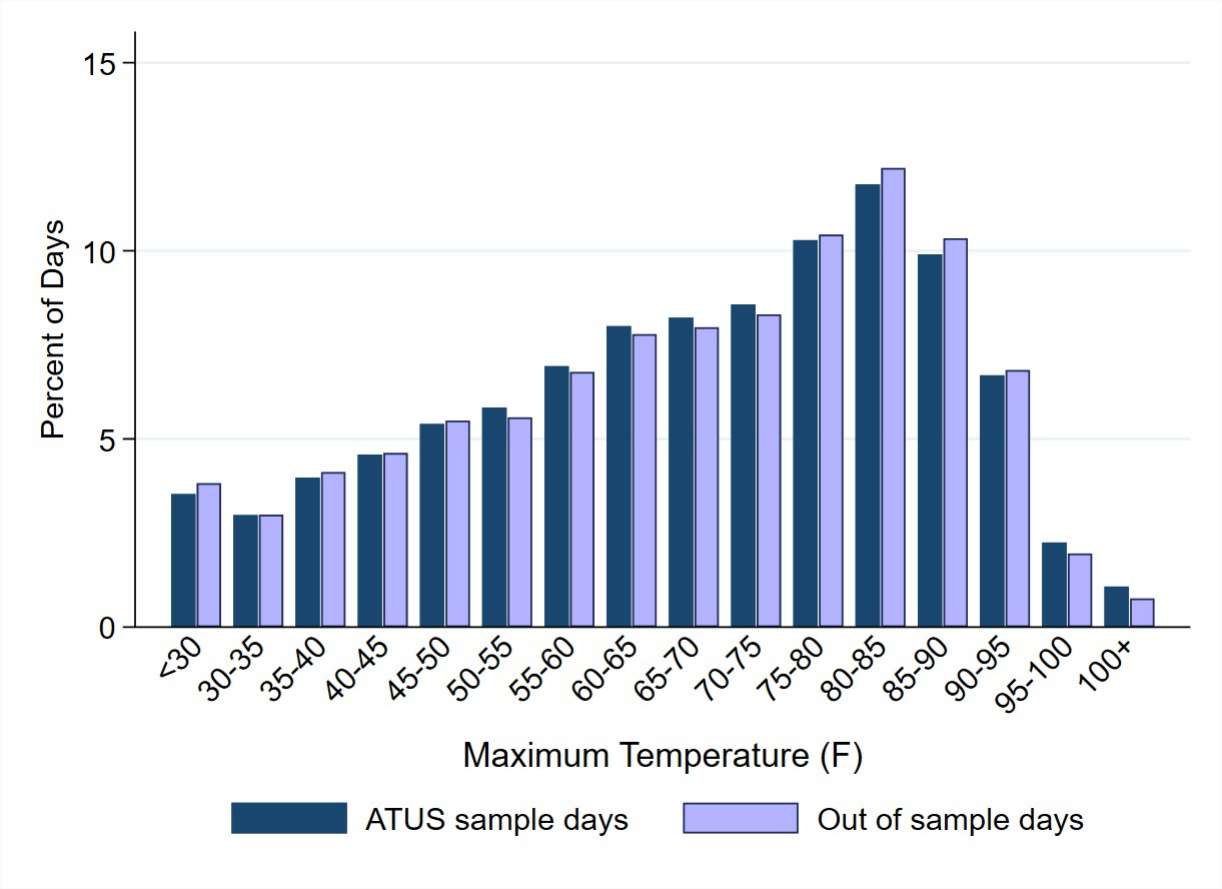
Historical climate. Distribution of daily maximum temperature in counties with ATUS respondents over 1) days with ATUS diary entries in the county (“ATUS sample days”), and 2) days without diary entries (“Out of sample days”).

We obtained the key weather variable, daily maximum temperature, as well as daily minimum temperature, precipitation, and snowfall for the study period from the Global Historical Climatology Network (GHCN)-Daily [9]. This dataset was built from various sources including the National Center for Environmental Information’s (NCEI) archive and the Global Summary of the Day collection, all of which have undergone consistent quality assurance procedures unique to the GHCN. Of the 100,000 stations available in the GHCN-Daily dataset, we draw from 46,114 stations for precipitation, 43,885 for snowmelt, and 10,717 for maximum and minimum temperature over the continental United States. Weather variables are averaged to the county level by day. In cases with no records in a county for a given day, we use the nearest station to the county centroid. Relative humidity is joined by county and date from PRISM [10]. There are 153,366 matched observations between ATUS responses and weather data by county and date. See Fig 1 for a summary of historical temperatures in ATUS sample counties.

We use the same methodology employed by GZN to identify sunset and sunrise times by county and date for all years before 2007. In 2007, daylight savings time was extended, so for all years subsequent to the change, we update the methodology to capture the shifted dates.

### Combined historical dataset

Across the sixteen-year period, the sample of available respondents with all data available includes 152,680 observations, among which 20,918 (14 percent) are employed in a high-risk industry and are included in our estimation sample. We split the dataset into three time periods: pre-recession (2003–2007), recession (2008–2014), and post-recession (2015–2018). Table 1 presents summary statistics for the full ATUS dataset, the sub-sample with location identified, and separately for high-risk workers by time period. The table showcases a slight reduction in labor hours during the recession period and an increase in hours worked in the post-recession period relative to the recession and pre-recession periods, both overall and conditional on working a given day. Among those that reported some work on their diary day, the average number of hours worked was 7.7. We also find that about 10 percent of all days included in the data set reach 90 degrees, with relatively similar percentages across the included time periods. The remaining 90 percent of days are split relatively evenly between the other two temperature segments.

**Table 1:** Summary statistics.

	All ATUS		ATUS with location		High-risk workers							
					All years		Pre-recession		Recession		Post-recession	
	(N = 201,151)		(N = 153,366)		(N = 20,918)		(N = 7,391)		(N = 9,186)		(N = 4,341)	
	Mean	SD	Mean	SD	Mean	SD	Mean	SD	Mean	SD	Mean	SD
Labor allocation												
Labor hours	2.64	4.00	2.67	4.00	4.40	4.53	4.41	4.50	4.36	4.53	4.47	4.58
Percent labor hours = 0	0.62	0.48	0.62	0.49	0.43	0.49	0.43	0.49	0.43	0.49	0.43	0.49
Labor hours if hours > 0	6.99	3.46	6.95	3.45	7.70	3.24	7.70	3.17	7.64	3.29	7.82	3.23
Maximum temp categories												
Min to 70 degrees F			0.49	0.50	0.51	0.50	0.52	0.50	0.51	0.50	0.48	0.50
70 to 90 degrees F			0.41	0.49	0.40	0.49	0.39	0.49	0.40	0.49	0.41	0.49
90 degrees F to max			0.10	0.30	0.10	0.29	0.09	0.28	0.10	0.30	0.10	0.31
Control variables												
Min temp (degrees F)			47.28	17.67	46.60	17.64	46.40	17.09	46.18	17.82	47.82	18.13
Precip. (inches/100)			11.30	30.17	11.31	30.43	11.12	29.17	11.20	30.33	11.90	32.68
Snow (inches/10)			0.67	5.24	0.61	4.76	0.60	4.30	0.66	5.38	0.51	4.02
Relative Humidity (%)			64.55	17.61	64.74	17.56	65.17	17.55	63.93	17.53	65.73	17.58
Sunrise (time)			6.43	0.50	6.44	0.51	6.60	0.63	6.35	0.41	6.35	0.41
Sunset (time)			18.57	1.24	18.56	1.24	18.71	1.36	18.48	1.17	18.50	1.16
Age	47.27	17.63	46.93	17.47	43.71	12.06	42.35	11.50	44.12	12.10	45.16	12.66
Over age 65	0.20	0.40	0.19	0.39	0.04	0.20	0.03	0.16	0.04	0.20	0.06	0.24
Male	0.44	0.50	0.44	0.50	0.75	0.44	0.74	0.44	0.75	0.43	0.75	0.44
Number of children < 18	0.85	1.14	0.86	1.14	0.99	1.17	1.05	1.18	0.98	1.17	0.90	1.16
Annual income ($1,000s)	48.09	65.44	51.73	68.91	90.78	70.59	82.91	63.63	91.11	70.85	103.51	78.94
Diary date a holiday	0.02	0.13	0.02	0.13	0.02	0.12	0.02	0.13	0.02	0.12	0.01	0.11
Employed	0.62	0.49	0.63	0.48	1.00	0.00	1.00	0.00	1.00	0.00	1.00	0.00
Absent from work	0.03	0.16	0.03	0.16	0.04	0.19	0.04	0.20	0.04	0.19	0.04	0.19
Out of labor force	0.33	0.47	0.32	0.47	0.00	0.00	0.00	0.00	0.00	0.00	0.00	0.00
Employed full time	0.49	0.50	0.50	0.50	0.90	0.30	0.91	0.29	0.89	0.31	0.89	0.31
White non-Hispanic	0.68	0.47	0.64	0.48	0.65	0.48	0.67	0.47	0.64	0.48	0.61	0.49
High school dropout	0.15	0.36	0.15	0.35	0.12	0.32	0.13	0.34	0.11	0.32	0.11	0.31
High school graduate	0.26	0.44	0.24	0.43	0.31	0.46	0.32	0.47	0.31	0.46	0.29	0.45
Some college	0.27	0.44	0.26	0.44	0.28	0.45	0.28	0.45	0.29	0.45	0.27	0.45
Spouse/partner in hh	0.53	0.50	0.52	0.50	0.64	0.48	0.67	0.47	0.64	0.48	0.61	0.49
NCA region, percent distribution												
Midwest	22%		20%		24%		23%		24%		23%	
Northeast	21%		23%		20%		23%		19%		18%	
Northern Great Plains	2%		1%		1%		1%		1%		1%	
Northwest	4%		4%		5%		5%		5%		5%	
Southeast	25%		22%		21%		18%		21%		24%	
Southern Great Plains	10%		10%		11%		9%		11%		12%	
Southwest	16%		20%		19%		21%		19%		18%	
Alaska and Hawaii	1%		0%		0%		0%		0%		0%	

Notes: Because the analysis relies on a non-random sub-sample of respondents, survey weights provided by ATUS are not employed in the analysis, including this table. Weather variables cannot be summarized for the full ATUS sample because location is required to match with this information.

These summary statistics also allow us to provide a characterization of the average high-risk worker in our sample. On average, the surveyed workers are between age 40 and 45; are 75 percent male; and are 65 percent white non-Hispanic. Nominal annual family incomes range from $82,000 to $104,000 across the reference time period and 90 percent are employed full time. About 28 percent have some college education and 64 percent have a spouse or partner in the household.

We also compare the demographic information and regional distribution of the full ATUS sample relative to the high-risk sub-sample to determine the extent to which our results remain nationally representative. Overall, we find that high-risk workers are younger, more likely to be male, less likely to be white non-Hispanic, and have higher incomes. Geographically, the full ATUS sample, ATUS sample with location information identified, and high-risk sub-sample are similarly distributed, suggesting that the presence of location information does not bias our sample towards respondents from specific regions. Demographic characteristics are relatively similar between the full ATUS sample and the ATUS sample with location information identified.

### Impact projections data

We use climate and socioeconomic projection data compatible with the Climate Change Impacts and Risk Analysis (CIRA) framework, which is designed to estimate and compare economic impacts across multiple sectors and regions of the United States [11, 12]. To estimate the time allocation and economic impacts of future climate, we employ several data sets. For future climate projections, we use a suite of six downscaled CMIP5 GCMs for RCP4.5 and RCP8.5 (See S1 Text for more information on climate models and scenarios). We calculate the degree days above 90 by era by first summing across all degrees above 90 by year for each county and averaging the annual number over the historical baseline (1986–2005) and four future eras: 2030 (2020–2039), 2050 (2040–2059), 2070 (2060–2079), and 2090 (2080–2099). Baseline era degree days are subtracted from each future era for the projections.

Information on the number of high-risk workers at the county level comes from the American Community Survey centered around 2010 (2008–2012 five-year estimates) [13]. We assume the number of high-risk workers will remain constant over time. This is because trends from the recent past, as well as near-term projections, suggest that while some industries defined as high-risk will reduce the number of workers they support, including agriculture and mining, and others have or will experience slight increases; on net the absolute number of high-risk workers has and is expected to remain roughly constant at least through 2029 [14].

An hourly wage rate specific to high-risk workers is constructed using average weekly wage rates and the average weekly hours worked in high-risk industries in 2009 from the Bureau of Labor Statistics [15, 16]. We find that the average high-risk worker allocated 40.5 hours to work per week, for a total of 2,108 hours per year. By weighting the industry-specific wages by the number of high-risk workers in each industry, we calculate an average hourly wage rate of $26.54 (2015 dollars). Future wage rates are adjusted using a per capita GDP adjustment factor, see S5 Table for details on population and economic growth projections [17].

This national-level data also demonstrates that the distribution of high-risk workers across industries in the United States is similar to the distribution we observe in the ATUS data. The Bureau of Labor Statistics data finds 5 percent of high-risk workers in the agriculture, forestry, and hunting sectors (relative to 4 percent of high-risk workers with locations in ATUS); 3 percent in mining, quarrying, and oil and gas extraction (1 percent in ATUS); 19 percent in utilities, transportation, and warehousing (22 percent in ATUS); 25 percent in construction (26 percent in ATUS); and 49 percent in manufacturing (47 percent in ATUS). This close match provides reassurance that drawing a sub-sample from the ATUS data still results in an industry-specific distribution well-aligned with national trends.

## Models and methods

### Historical relationship between temperature and labor time

Our econometric model mirrors that of GZN and includes county fixed effects, which enables us to identify the effects of temperature using the plausibly exogenous variation in temperature over time within counties. This identification strategy has also been employed in other studies examining various aspects of climate change [18, 19]. While the focus of our investigation is on time devoted to labor, the time allocated to non-work time spent outdoors and indoors are jointly determined. Following GZN, we estimate a system of equations to examine the relationship between maximum daily temperature *temp* and time (minutes) allocated to labor *labor* for individual *i* in county *c* on date *t*:

$${labor}_{ict}=\beta_{1}+\gamma_{1}f_{1}({temp}_{ct})+{\delta_{1}X}_{ict}+{\theta_{1}Z}_{ct}+\alpha_{1c}+g_{1}(time)+\epsilon_{1ict}$$

$${outdoor}_{ict}=\beta_{2}+\gamma_{2}f_{2}({temp}_{ct})+{\delta_{2}X}_{ict}+{\theta_{2}Z}_{ct}+\alpha_{2c}+g_{2}(time)+\epsilon_{2ict}$$

$${indoor}_{ict}=\beta_{3}+\gamma_{3}f_{3}({temp}_{ct})+{\delta_{3}X}_{ict}+{\theta_{3}Z}_{ct}+\alpha_{3c}+g_{3}(time)+\epsilon_{3ict}$$

We estimate these equations simultaneously via non-linear least squares regression. This method allows us to constrain the time allocation changes to sum to zero across the three time-use categories [20].

Our model differs from GZN in two ways. First, *temp* is represented here using a linear spline with knots at 70 degrees and 90 degrees, creating three separate sections of a piece-wise linear function. This approach improves the efficiency of estimates relative to the binned approach in the original analysis. These knots were chosen based on the patterns in GZN. In particular, none of the bins below the omitted 76 to 80 degree control category were statistically significant, suggesting that the observations at the low end of the distribution could be combined into one linear segment. Statistically significant differences emerged in temperature bins over 90 degrees and displayed a linear pattern beyond that, suggesting a knot at that location would be appropriate.

Second, to control for time trends, we include natural cubic splines (with four knots based on Harrell’s (2015) recommended percentiles [21]) specific to each year, denoted by the variable *time*. The original study controlled for the effects of time using month-year dummy variables, which assumes a constant relationship within each month that discretely changes at the beginning of each new month. The use of splines allows us to control for seasonal differences specific to each year while maintaining a continuous relationship in our outcomes within a given year. It also improves efficiency by reducing the number of estimated parameters.

As in GZN, the vector *X* includes control variables at the individual level while the vector *Z* includes other weather and environmental variables at the county-date level, as summarized in Table 1. We control for unobserved and time-constant county characteristics using county-level fixed effects *α* (accomplished by de-meaning all variables and observations within a given county). Standard errors are clustered at the state-month level to control for autocorrelation within counties and spatial correlation across countries within states.

Due to the significant shocks to the labor market during the Great Recession, we estimate the model separately for three time periods: pre-recession (2003–2007), recession (2008–2014), and post-recession (2015–2018). We also run the model combining all time periods and a combined pre- and post-recession period.

### Simulated future impacts of climate change on labor time

To estimate the impact of future high temperature days on time allocated to labor per high-risk worker, we use the modeled coefficient estimate *γ*_1_ coupled with future climate projection data for each era 2030, 2050, 2070, and 2090, defined as 20-year eras relative to a 1995 baseline. We calculate the per worker time impacts relative to observed work hours in 2009 from the Bureau of Labor Statistics. To estimate the economic impact at the county and national level, we scale the per worker estimates using the number of high-risk workers in a given county in 2010 and calculated average wage rates.

## Results

### Historical relationship between temperature and labor time

Table 2 presents the coefficient estimates for *γ*_1_ as well as standard errors for the labor model specifically. It demonstrates that across the full 2003 to 2018 period, there is no statistically significant change in time allocated to labor on days above 90 degrees. However, focusing solely on the periods of economic growth (2003–2007 and 2015–2018) shows a statistically significant negative effect of high temperature days on time dedicated to labor. The pre- and post-recession periods are combined to increase sample size, and thus power, though the coefficient estimates in the two individual periods support the same conclusions. The coefficient for the pre- and post-recession period indicates that for every degree above 90 on a particular day, the average high-risk worker reduces their time devoted to work by about 2.6 minutes relative to a 90-degree day. In other words, on a day reaching 100 degrees, high-risk workers reduce their work time by 26 minutes ((100–90) * 2.6) relative to a 90-degree day. Based on an 8-hour workday, this reflects a 5.4 percent decrease in hours worked. These results hold when manufacturing is dropped from the high-risk category, when industry controls are included, and when the model is run without covariates (see S2–S4 Tables).

**Table 2:** Estimation results: All years and by recession/non-recession periods.

	Pre-recession (N = 7,391)	Recession (N = 9,186)	Post-recession (N = 4,341)	Pre- and post-recession (N = 11,732)	All years (N = 20,918)
Min to 70 degrees	-0.204	-0.165	0.311	-0.052	-0.052
	0.418	0.382	0.555	0.332	0.251
70 to 90 degrees	-0.117	-0.584	-0.508	-0.359	-0.412
	0.656	0.571	0.792	0.497	0.373
90 degrees to max	-2.653	0.389	-1.995	-2.595	-1.047
	1.583	1.364	1.997	1.239	0.923
	*			**	

Notes: Results of labor model only. Coefficient estimates in first row followed by standard errors clustered at the state-month level.

* denotes statistical significance at the 90^th^ percentile while

** denotes statistical significance at the 95^th^ percentile. Estimation sample includes only high-risk workers.

Furthermore, the model does not suggest any strong relationship between temperature and labor allocation at lower temperature levels. This is true for both moderate temperatures (70–90 degrees) as well as cooler temperatures (min to 70 degrees). For example, the estimate of -.05 for the lowest temperature category, although statistically insignificant, would imply a two-minute decrease in time worked when shifting from 70 to 30 degrees ((70–30) * -.05). This finding further justifies our focus on the highest temperature days.

Table 3 presents the results of a set of simplified linear regressions to demonstrate the robustness of the estimated effect on days over 90 degrees. In these regressions, we control for industry, year, county, time of year (defined either by summer/non-summer or four seasons), and recession period (2008–2014), along with the same set of demographic and weather controls included in the main specification. Standard errors are clustered at the state-month level. The effect of temperature on time allocated to labor by high-risk workers is not significant when the recession is modeled by an indicator variable alone and seasonality is controlled for as four seasons. When we allow the effect to vary within and outside of the recession by interacting temperature and the recession period, the results suggest high-risk workers allocate 5.3 to 5.9 less minutes per day per degree of above 90 to labor outside of the recession period (compared to 2.6 minutes in the preferred specification). During the recession, however, the interaction term neutralizes this effect. Although not significant at the 90 percent level, the p-values on the variables of interest in the two models of high-risk workers with interaction terms (i.e., maximum temperature, recession, and the interaction term) have p-values less than 0.122. Finally, we run the same specification for low-risk workers and find this effect is limited to high-risk workers, matching the findings in GZN.

**Table 3:** Linear regression results restricted to days over 90 degrees.

	High risk No interaction Season control	High risk Interaction Season control	High risk Interaction Summer control	Low risk Interaction Summer control
	(N = 1,997)	(N = 1,997)	(N = 1,997)	(N = 7,315)
Max Temp	-2.833	-5.316	-5.908	-0.147
	2.858	3.250	3.247	1.542
			*	
Recession = 1	3.234	-504.588	-511.964	156.528
	46.822	324.772	326.870	133.143
Max Temp x Recession		5.302	5.383	-1.363
		3.405	3.424	1.370
Season = 2	-81.091	-80.637		
*(Mar-May)*	84.336	84.379		
Season = 3	-122.673	-122.507		
*(Jun-Aug)*	87.193	87.336		
Season = 4	-136.823	-137.199		
*(Sep-Nov)*	77.217	77.071		
	*	*		
Summer = 1			61.044	-14.480
*(Apr-Sep)*			42.285	22.505

Notes: Estimates of effects on minutes allocated to labor per day. Estimates in first row followed by standard errors clustered at the state-month level.

* denotes statistical significance at the 90th percentile.

### Simulated future impacts of climate change on labor time

We apply the results from the combined pre- and post-recession time period to estimate the future effects of climate on labor time allocation and the analogous economic impacts. We adjust the pre- and post-recession model estimate by the expected proportion of time the economy may be in a period of expansion for the remainder of the 21^st^ century based on observed cycles over the past 50 years (86.2 percent over January 1970 through December 2019, inclusive) [22].

**Fig 2:**
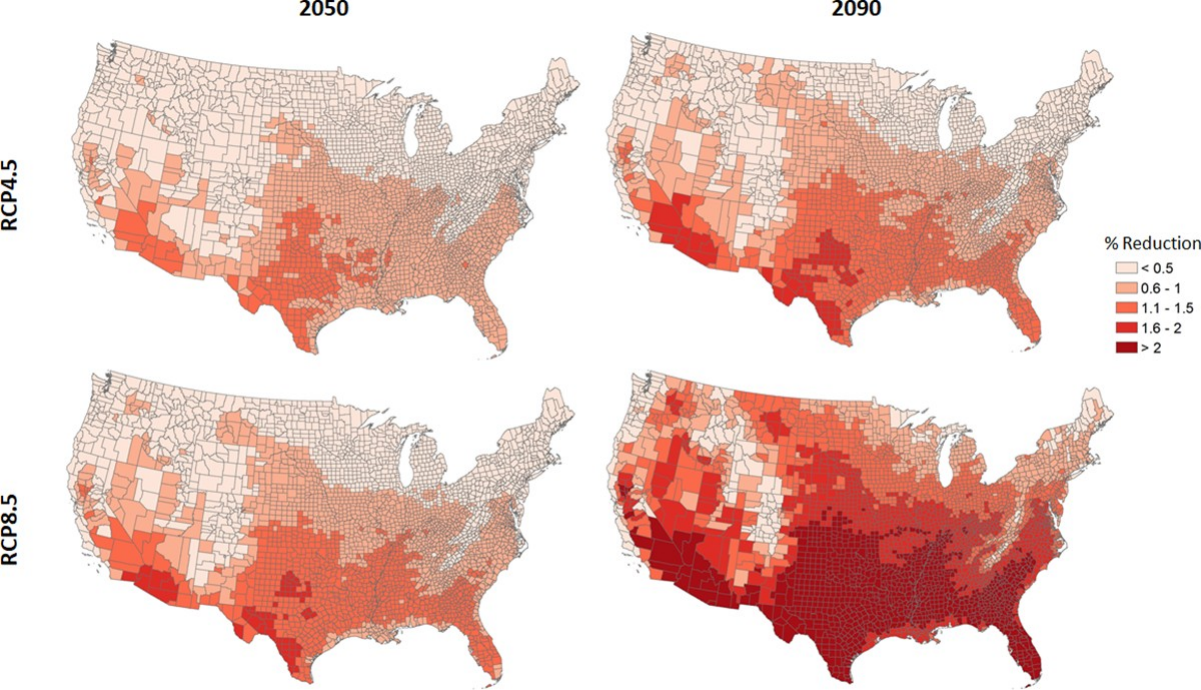
Projected percent change in future labor hours per worker. Percent reductions relative to a 40.5-hour work week, as observed among high-risk workers using data from the Bureau of Labor Statistics in 2009. These results represent an average across the six GCMs.

Fig 2 presents the percent reduction in labor time per worker relative to time worked in 2009 (40.5 hours on average) by county under the 2050 and 2090 scenarios for both RCP 4.5 and 8.5 relative to the 1995 climate baseline. The differences across counties reflect differences in projected temperatures, not differences in number or concentration of high-risk workers. This figure illustrates high-risk workers in large parts of the country could experience greater than 2 percent decreases in total labor hours on account of the increase in degrees over 90 by 2090, especially under the higher emissions RCP 8.5 scenario. The largest percent reduction in time per worker at the county level is about 4.1 percent under the RCP 8.5 scenario in 2090. Few areas of the country appear to be spared from an increase in number of degree days over 90, resulting in less than 0.5 percent decreases in time worked throughout the 21^st^ century, including but not limited to northern and coastal Maine, coastal Pacific Northwest, and some areas of the Rocky Mountains.

Table 3 translates these per worker impacts to the national level. On average across the country, the percent reduction in time worked per high-risk worker approaches 1.5 percent in 2090 relative to actual hours worked in 2009 under RCP8.5. The RCP 4.5 and 8.5 scenarios provide relatively similar estimates in 2030 (0.32 percent and 0.36 percent, respectively) but diverge considerably by 2090 (0.68 percent and 1.49 percent, respectively).

Table 4 further explores how these per-worker estimates could translate into national level economic impacts. It values all lost labor time by applying the national-level weighted-average wage rate. This approach assumes that the observed wage rate is a good proxy for lost economic productivity on account of reduced work time. Furthermore, it applies the per-worker estimates to all high-risk workers counted in 2009. We find that annual lost wages resulting from decreased time spent working on days over 90 degrees could be as high as $8.7 billion in 2030, $21.0 billion in 2050, $45.1 billion in 2070, and $80.0 billion in 2090 under the higher emissions RCP 8.5 scenario (2015 dollars). Under the lower emissions RCP 4.5 scenario, lost wages could reach $7.8 billion in 2030, $15.8 billion in 2050, $25.8 billion in 2070, and $36.7 billion in 2090 (2015 dollars).

**Table 4:** National-level projections of lost labor time and lost wages due to climate change.

	National average percent reduction in work time per worker relative to 2009 hours worked		National total lost wages on account of time lost across all high-risk workers (2015 billion dollars)	
Era	RCP 4.5	RCP 8.5	RCP 4.5	RCP 8.5
2030	0.32	0.36	$7.8	$8.7
2050	0.48	0.64	$15.8	$21.0
2070	0.61	1.06	$25.8	$45.1
2090	0.68	1.49	$36.7	$80.0

Notes: All values represent averages across the 6 GCMs. Climate baseline is 1995 era. Wage rate and hours worked baseline is 2009. Time lost relative to a 90-degree day (different reference point than GZN). Based on the pre- and post- recession model results, adjusted based on proportion of observed expansion over the last 50 years (86.2 percent).

The use of climate and socioeconomic scenarios from the CIRA framework allows for the comparison of economic impacts estimated in this study to other sectoral damages estimated for the United States. Compared with more than 20 other modeled sectors for 2090 damages under RCP 8.5, these projected lost wages ($80.0 billion per year) rank third, with extreme temperature mortality ($140 billion per year) and coastal property damages ($120 billion per year) showing larger economic risk [12].

Comparing our results to previous labor allocation findings [23] that were based on the original GZN approach, we find that annual lost wages in 2090 under RCP 8.5 are approximately half as large. The primary drivers of this difference are that the previous approach and findings scaled the number of high-risk workers over time in proportion to population growth (we hold this constant) and were based on trajectories of economic growth that did not adjust for the likelihood and effects of future recession periods (we apply the results only to periods of economic growth). See S2 Text for additional detail. Regardless, the magnitude of economic costs presented in this labor allocation study underscores the importance of including this sector when evaluating policies to address climate change.

## Conclusion

As global temperatures continue to warm, impacts on our daily lives will increase substantially. Understanding how individuals respond to these changes is essential for the design of well-formulated policies. In this paper, we examine the impacts of temperature on individuals’ allocation of time to work within the United States over an extended 16-year period. Setting aside the period of the Great Recession, our results are strikingly similar to those found in seminal work by GZN, whose focus was limited to a brief 4-year window, providing additional evidence on the generalizability of the harmful impacts of extreme heat on labor markets in exposed sectors and highlighting its importance for consideration by decisionmakers.

At the same time, the absence of any relationship between extreme temperature and hours worked during the Great Recession offers insights into the mechanisms behind the above finding, suggesting that supply-side factors drive this relationship. Workers adjust their labor supply in response to temperature extremes during strong labor market conditions but continue to work at comparable rates during weak labor market conditions as temperatures rise, perhaps because they face increased job security concerns during the recessionary period or because employers are less flexible about granting heat-related leave during difficult economic times.

This pattern of results also highlights the context-specific nature of impacts. Since labor responses appear to depend on the ebb and flow of business cycles, this logic may also apply across industries and countries depending on the strength of labor laws. Sectors that offer workers greater job security may experience larger responses to temperature extremes while those with fewer protections may experience more muted responses.

These more muted responses, however, do not necessarily imply an absence of labor market impacts from hotter temperatures. Workers may supply labor at the same rates across the temperature distribution because of job security concerns, but these hours are likely to be costlier to supply under adverse environmental conditions. Whether these costs are largely borne by workers who must simply supply more effort to overcome adverse environmental conditions or employers who find their workers are less productive while on the job is an open question and an important area for future research, as we do not estimate these effects. For example, despite supplying the same amount of labor, workers may experience heat-related symptoms that increase health risks or lead to fatigue that may diminish their effectiveness on the job. Regardless, the absence of effects on the extensive margin of labor hours worked during economic contractions does not imply that extreme heat is costless in those settings.

Despite the strengths of our methodology, we note some important limitations. First, there may be important substitutions in work schedules within days that our analysis does not capture. GZN find evidence of comparable start times but early end times, which suggests this concern is likely to be limited in this setting. Second, given the long time frame of the analysis, workers may migrate depending on local climate and economic conditions. Our use of county fixed effects (FEs) helps to mitigate this concern to some extent because our estimates are based on variations in temperature and time allocation within a county. Migration would introduce a bias if people choose a county based on the variation in temperature in the county and they have a differential response to temperature compared to those who stay. Since our data reflects a repeated cross-section rather than a longitudinal data set, unfortunately we cannot assess the degree to which this exists.

To place our results in a broader policy context, we use our estimates from the non-recessionary period to generate estimates of labor hours and wages lost due to climate change. We find that the average high-risk worker could decrease time allocated to labor by 1.5 percent in 2090 relative to actual hours worked in 2009 under the higher emissions RCP 8.5 scenario. This would translate to lost wages of $80 billion across all high-risk workers in the continental United States (2015 dollars). Should the future hold more significant recessions, projected labor impacts will be smaller, but total welfare losses will remain high. As such, our results provide further evidence of increasing damages in a warming climate, and justification for efforts to reduce emissions. The results can also help to diffuse arguments between business interests and environmentalists by highlighting one important channel through which environmental protection can help to enhance the financial health of firms.

That said, the magnitude of the climate impacts should be treated with some caution, as these forecasts are predicated on the assumption that the effect of temperature on hours worked remains constant over time. Given the long timeframe over which climate change occurs, workers and firms may adapt to warmer temperatures in a way that fundamentally alters this relationship in the future. There are likely heterogenous opportunities to adapt across the industries included in the high-risk categorization; agricultural workers, who make up a small percentage of the sample, may be particularly limited in their abilities to adapt due to the nature of their work, while it may be easier for manufacturing facilities or manufacturing workers, who dominate the sample, to adapt by improving climate control technologies in their facilities. These adaptations, however helpful in muting the response, are likely to be partial at best (e.g., due to technological limitations, uncertainty about the future, and local factors that make adaptation infeasible) and may be costly to undertake. At the same time, our focus on high-risk sectors ignores longer-run labor-market spillover effects to less climate-exposed industries that could arise through the reallocation of talent within the labor market, or spillover effects in related industries. Any comprehensive accounting of the impacts of climate change on firms and labor markets would need to attend to these adaptation costs and spillover effects as they arise.

## Supporting information

S1 Table Full regression results. (DOCX)

S2 Table Regression results, excluding manufacturing as a high-risk industry. (DOCX)

S3 Table Regression results, with industry controls. (DOCX)

S4 Table Regression results, no covariates. (DOCX)

S5 Table Sources of data for this analysis. (DOCX)

S1 Text Climate projection models and scenarios. (DOCX)

S2 Text Comparison to previous estimate (Martinich and Crimmins, 2019). (DOCX)
