# Supplementary material for: Temperature and work: Time allocated to work under varying climate and labor market conditions
Source: PLoS One. 2021 Aug 25;16(8):e0254224. doi: 10.1371/journal.pone.0254224 (PMC8386856; doi:10.1371/journal.pone.0254224)
Supplement: S1 Table — (DOCX) [file pone.0254224.s001.docx]

**S1 Table. Full Regression Results**

|  | Pre-recession | Recession | Post-recession | Pre- and post- recession | | All years |
| --- | --- | --- | --- | --- | --- | --- |
|  | (n=3,847) | (n=4,966) | (n=2,308) | (n=6,155) | | (n=11,121) |
| Constant | 0.000 | 0.000 | 0.000 | 0.000 | | 0.000 |
|  | 2.231 | 2.038 | 2.875 | 1.780 | | 1.358 |
|  |  |  |  |  | |  |
| Min to 70 degrees | -0.204 | -0.165 | 0.311 | -0.052 | | -0.052 |
|  | 0.418 | 0.382 | 0.555 | 0.332 | | 0.251 |
|  |  |  |  |  | |  |
| 70 to 90 degrees | -0.117 | -0.584 | -0.508 | -0.359 | | -0.412 |
|  | 0.656 | 0.571 | 0.792 | 0.497 | | 0.373 |
|  |  |  |  |  | |  |
| 90 degrees to max | -2.653 | 0.389 | -1.995 | -2.595 | | -1.047 |
|  | 1.583 | 1.364 | 1.997 | 1.239 | | 0.923 |
|  | * |  |  | ** | |  |
| Min temp (degrees F) | 0.159 | 0.458 | -0.454 | 0.023 | | 0.197 |
|  | 0.472 | 0.418 | 0.613 | 0.369 | | 0.277 |
|  |  |  |  |  | |  |
| Precip. (inches/100) | 0.070 | -0.025 | -0.011 | 0.028 | | 0.004 |
|  | 0.099 | 0.070 | 0.131 | 0.078 | | 0.053 |
|  |  |  |  |  | |  |
| Snow (inches/10) | 0.326 | -0.221 | 0.873 | 0.502 | | -0.005 |
|  | 0.517 | 0.318 | 0.719 | 0.413 | | 0.271 |
|  |  |  |  |  | |  |
| Relative Humidity (%) | 0.181 | -0.222 | -0.196 | 0.017 | | -0.084 |
|  | 0.174 | 0.186 | 0.262 | 0.144 | | 0.115 |
|  |  |  |  |  | |  |
| Sunrise (time) | 5.645 | -3.020 | -3.243 | 3.542 | | 2.322 |
|  | 5.985 | 6.851 | 10.008 | 4.679 | | 3.725 |
|  |  |  |  |  | |  |
| Sunset (time) | -2.590 | -9.487 | -13.878 | -6.536 | | -8.243 |
|  | 5.086 | 5.417 | 8.255 | 4.074 | | 3.158 |
|  |  | * | * |  | | *** |
| DOW: Mon | 347.592 | 362.292 | 359.358 | 351.318 | | 356.433 |
|  | 9.095 | 7.793 | 11.641 | 7.101 | | 5.261 |
|  |  |  |  |  | |  |
| DOW: Tues | 371.295 | 365.839 | 379.370 | 376.294 | | 370.491 |
|  | 8.445 | 7.549 | 11.307 | 6.642 | | 4.948 |
|  |  |  |  |  | |  |
| DOW: Wed | 373.291 | 368.723 | 386.812 | 378.252 | | 374.435 |
|  | 8.179 | 7.621 | 10.726 | | 6.453 | 4.910 |
|  |  |  |  | |  |  |
| DOW: Thurs | 376.962 | 371.801 | 386.088 | | 382.245 | 376.875 |
|  | 8.691 | 7.444 | 11.032 | | 6.726 | 4.927 |
|  |  |  |  | |  |  |
| DOW: Fri | 348.183 | 328.839 | 332.669 | | 341.067 | 334.491 |
|  | 8.868 | 8.443 | 12.830 | | 7.259 | 5.501 |
|  |  |  |  | |  |  |
| DOW: Sat | 42.476 | 34.977 | 38.555 | | 41.050 | 39.047 |
|  | 6.611 | 5.953 | 8.647 | | 5.162 | 3.886 |
|  |  |  |  | |  |  |
| Age | 0.237 | -0.246 | -0.063 | | 0.118 | -0.022 |
|  | 0.216 | 0.201 | 0.288 | | 0.171 | 0.129 |
|  |  |  |  | |  |  |
| Male | 27.144 | 32.955 | 30.386 | | 28.812 | 30.043 |
|  | 4.981 | 4.509 | 7.309 | | 4.089 | 3.037 |
| Number of children < 18 | -1.771 | -4.989 | -4.346 | | -2.572 | -3.857 |
|  | 2.276 | 2.063 | 3.012 | | 1.806 | 1.352 |
|  |  | ** |  | |  | *** |
| Annual income ($1,000s) | 0.000 | 0.000 | 0.000 | | 0.000 | 0.000 |
|  | 0.000 | 0.000 | 0.000 | | 0.000 | 0.000 |
|  |  |  |  | |  |  |
| Diary date a holiday | -181.601 | -226.977 | -193.519 | | -179.853 | -200.034 |
|  | 20.825 | -21.231 | 32.916 | | -17.293 | -13.394 |
|  |  |  |  | |  |  |
| Employed | - | - | - | | - | - |
|  | - | - | - | | - | - |
|  |  |  |  | |  |  |
| Absent from work | -212.214 | -204.321 | -187.232 | | -204.591 | -203.717 |
|  | -11.255 | -10.093 | -18.595 | | -9.483 | -6.903 |
|  |  |  |  | |  |  |
| Out of labor force | - | - | - | | - | - |
|  | - | - | - | | - | - |
|  |  |  |  | |  |  |
| Employed full time | 122.467 | 122.757 | 116.299 | | 119.709 | 122.263 |
|  | 8.611 | 7.224 | 11.489 | | 6.756 | 4.931 |
|  |  |  |  | |  |  |
| White non-Hispanic | -9.991 | -6.687 | 3.114 | | -4.868 | -5.726 |
|  | 6.266 | 5.349 | 7.628 | | 4.718 | 3.516 |
|  |  |  |  | |  |  |
| High school dropout | -10.815 | -11.146 | -12.472 | | -12.001 | -10.908 |
|  | 9.379 | 8.518 | 12.441 | | 7.373 | 5.484 |
|  |  |  |  | |  | ** |
| High school graduate | 6.128 | -3.179 | -13.117 | | -1.742 | -2.867 |
|  | 7.103 | 6.124 | 9.561 | | 5.597 | 4.109 |
|  |  |  |  | |  |  |
| Some college | 1.265 | 8.681 | 5.858 | | 1.669 | 4.625 |
|  | 6.787 | 5.880 | 8.951 | | 5.323 | 3.928 |
|  |  |  |  | |  |  |
| Spouse/partner in hh | 2.078 | -1.574 | 2.889 | | 1.601 | 0.848 |
|  | 5.623 | 5.094 | 6.997 | | 4.347 | 3.290 |
|  |  |  |  | |  |  |
| Family income less than $5,000 | 58.381 | -15.264 | -67.605 | | 18.766 | 12.215 |
|  | 23.671 | 24.816 | 25.200 | | 18.692 | 15.315 |
|  | ** |  | *** | |  |  |
| Family income $5,000 to $7,499 | 22.953 | -41.533 | 0.125 | | 29.658 | 2.560 |
|  | 28.609 | 23.280 | 34.518 | | 22.701 | 16.196 |
|  |  | * |  | |  |  |
| Family income $7,500 to $9,999 | 16.281 | 2.622 | -21.751 | | 16.707 | 15.246 |
|  | 24.960 | 23.443 | . | | 20.478 | 15.045 |
|  |  |  |  | |  |  |
| Family income $10,000 to $12,499 | -2.991 | -16.267 | 4.130 | | 6.426 | 5.062 |
|  | 22.744 | 21.047 | 23.394 | | 17.060 | 13.286 |
|  |  |  |  | |  |  |
| Family income $12,500 to $14,999 | 8.082 | -47.285 | -23.942 | | 13.892 | -11.254 |
|  | 20.747 | 20.447 | 35.679 | | 17.949 | 13.413 |
|  |  | ** |  | |  |  |
| Family income $15,000 to $19,999 | 5.800 | -13.419 | -26.095 | | 9.211 | 2.273 |
|  | 17.123 | 17.489 | 22.388 | | 14.318 | 10.909 |
|  |  |  |  | |  |  |
| Family income $20,000 to $24,999 | 13.188 | 3.173 | -7.788 | | 16.437 | 16.175 |
|  | 13.426 | 15.728 | 16.571 | | 11.212 | 9.079 |
|  |  |  |  | |  | * |
| Family income $25,000 to $29,999 | 18.032 | -29.222 | -32.451 | | 11.474 | -1.948 |
|  | 12.305 | 14.347 | 15.466 | | 10.409 | 8.407 |
|  |  | ** | ** | |  |  |
| Family income $30,000 to $34,999 | -2.308 | -28.863 | -23.927 | | 2.726 | -7.282 |
|  | 11.557 | 13.681 | 19.076 | | 10.495 | 8.145 |
|  |  | ** |  | |  |  |
| Family income $35,000 to $39,999 | -16.781 | -33.631 | -28.522 | | -9.966 | -15.311 |
|  | 12.846 | 14.055 | 15.920 | | 10.870 | 8.419 |
|  |  | ** | * | |  | * |
| Family income $40,000 to $49,999 | -8.145 | -21.140 | -19.750 | | -1.902 | -5.475 |
|  | 10.599 | 12.901 | 13.047 | | 9.162 | 7.378 |
|  |  |  |  | |  |  |
| Family income $50,000 to $59,999 | -0.213 | -14.770 | -34.126 | | -2.392 | -4.154 |
|  | 10.388 | 12.355 | 12.466 | | 9.026 | 7.104 |
|  |  |  | *** | |  |  |
| Family income $60,000 to $74,999 | 2.269 | -21.649 | -25.325 | | 2.591 | -4.100 |
|  | 9.530 | 12.127 | 12.672 | | 8.477 | 6.861 |
|  |  | * | ** | |  |  |
| Family income $75,000 to $99,999 | 11.448 | -17.611 | -16.400 | | 12.753 | 2.817 |
|  | 8.655 | 11.702 | 11.664 | | 8.015 | 6.536 |
|  |  |  |  | |  |  |
| Family income $100,000 to $149,999 | 6.871 | -16.078 | -35.746 | | 0.598 | -1.462 |
|  | 9.940 | 11.865 | 11.892 | | 8.600 | 6.844 |
|  |  |  | *** | |  |  |
| Family income $150,000 and over | 8.681 | -13.911 | -27.632 | | 3.805 | -0.105 |
|  | 12.985 | 12.466 | 13.198 | | 9.901 | 7.489 |
|  |  |  | ** | |  |  |
| R-squared | 0.470 | 0.464 | 0.474 | | 0.470 | 0.464 |
| Notes: Results of labor model only. Coefficient estimates in first row followed by standard errors clustered at the state-month level. * denotes statistical significance at the 90^th^ percentile, ** denotes statistical significance at the 95^th^ percentile, and *** denotes statistical significance at the 99^th^ percentile. Spline coefficient estimates not shown due to space requirements. | | | | | | |
