# Supplementary material for: Temperature and work: Time allocated to work under varying climate and labor market conditions
Source: PLoS One. 2021 Aug 25;16(8):e0254224. doi: 10.1371/journal.pone.0254224 (PMC8386856; doi:10.1371/journal.pone.0254224)
Supplement: S2 Table — (DOCX) [file pone.0254224.s002.docx]

**S2 Table. Regression Results, Excluding Manufacturing as a High-Risk Industry**

|  | Pre-recession  (N=3,847) | Recession  (N=4,966) | Post-recession  (N=2,308) | Pre- and post-recession  (N=6,155) | All years  (N=11,121) |
| --- | --- | --- | --- | --- | --- |
| Min to 70 degrees | -0.632 | 0.201 | 0.382 | -0.365 | -0.033 |
|  | 0.624 | 0.545 | 0.834 | 0.478 | 0.360 |
|  |  |  |  |  |  |
| 70 to 90 degrees | 0.499 | 0.527 | -0.874 | -0.239 | 0.001 |
|  | 0.957 | 0.853 | 1.17 | 0.711 | 0.542 |
|  |  |  |  |  |  |
| 90 degrees to max | -4.038 | -0.796 | -2.140 | -3.608 | -1.873 |
|  | 2.414 | 1.826 | 2.606 | 1.704 | 1.229 |
|  | * |  |  | ** |  |
| Notes: Results of labor model only. Coefficient estimates in first row followed by standard errors clustered at the state-month level. * denotes statistical significance at the 90^th^ percentile while ** denotes statistical significance at the 95^th^ percentile. Estimation sample includes only high-risk workers, excluding manufacturing. | | | | | |
