# Supplementary material for: Temperature and work: Time allocated to work under varying climate and labor market conditions
Source: PLoS One. 2021 Aug 25;16(8):e0254224. doi: 10.1371/journal.pone.0254224 (PMC8386856; doi:10.1371/journal.pone.0254224)
Supplement: S3 Table — (DOCX) [file pone.0254224.s003.docx]

**S3 Table. Regression Results, with Industry Controls**

|  | Pre-recession  (N=7,391) | Recession  (N=9,186) | Post-recession  (N=4,341) | Pre- and post-recession  (N=11,732) | All years  (N=20,918) |
| --- | --- | --- | --- | --- | --- |
| Min to 70 degrees | -0.180 | -0.195 | 0.343 | -0.023 | -0.052 |
|  | 0.421 | 0.383 | 0.560 | 0.333 | 0.252 |
|  |  |  |  |  |  |
| 70 to 90 degrees | -0.071 | -0.569 | -0.427 | -0.290 | -0.371 |
|  | 0.660 | 0.572 | 0.797 | 0.498 | 0.373 |
|  |  |  |  |  |  |
| 90 degrees to max | -2.497 | -0.280 | -2.048 | -2.542 | -1.052 |
|  | 1.584 | 1.362 | 1.999 | 1.235 | 0.918 |
|  |  |  |  | ** |  |
| Notes: Results of labor model only. Coefficient estimates in first row followed by standard errors clustered at the state-month level. * denotes statistical significance at the 90^th^ percentile while ** denotes statistical significance at the 95^th^ percentile and *** denotes statistical significance at the 99^th^ percentile. Estimation sample includes only high-risk workers. High-risk industries include agriculture and forestry, mining, construction, manufacturing, and transport and utilities. | | | | | |
