# Supplementary material for: Temperature and work: Time allocated to work under varying climate and labor market conditions
Source: PLoS One. 2021 Aug 25;16(8):e0254224. doi: 10.1371/journal.pone.0254224 (PMC8386856; doi:10.1371/journal.pone.0254224)
Supplement: S4 Table — (DOCX) [file pone.0254224.s004.docx]

**S4 Table. Regression Results, No Covariates**

|  | Pre-recession  (N=7,391) | Recession  (N=9,186) | Post-recession  (N=4,341) | Pre- and post-recession  (N=11,732) | All years  (N=20,918) |
| --- | --- | --- | --- | --- | --- |
| Min to 70 degrees | -0.207 | 0.063 | -0.207 | -0.053 | 0.018 |
|  | 0.259 | 0.253 | 0.259 | 0.215 | 0.164 |
|  |  |  |  |  |  |
| 70 to 90 degrees | 0.336 | -0.044 | 0.336 | 0.211 | 0.045 |
|  | 0.528 | 0.491 | 0.528 | 0.415 | 0.319 |
|  |  |  |  |  |  |
| 90 degrees to max | -3.845 | -0.178 | -3.845 | -4.489 | -2.366 |
|  | 2.024 | 1.638 | 2.024 | 1.595 | 1.144 |
|  | ** |  | ** | *** | ** |
| Notes: Results of labor model only. Coefficient estimates in first row followed by standard errors clustered at the state-month level. * denotes statistical significance at the 90^th^ percentile while ** denotes statistical significance at the 95^th^ percentile and *** denotes statistical significance at the 99^th^ percentile. Estimation sample includes only high-risk workers. | | | | | |
