# Supplementary material for: Temperature and work: Time allocated to work under varying climate and labor market conditions
Source: PLoS One. 2021 Aug 25;16(8):e0254224. doi: 10.1371/journal.pone.0254224 (PMC8386856; doi:10.1371/journal.pone.0254224)
Supplement: S5 Table — (DOCX) [file pone.0254224.s005.docx]

**S5 Table. Sources of Data for this Analysis**

| **Data Type** | **Description** | **Data Documentation and Availability** |
| --- | --- | --- |
| Observed meteorology | Historical climate data for temperature, precipitation, and other weather variables. | Menne, M.J., I. Durre, B. Korzeniewski, S. McNeal, K. Thomas, X. Yin, S. Anthony, R. Ray, R.S. Vose, B.E.Gleason, and T.G. Houston, 2012: Global Historical Climatology Network - Daily (GHCN-Daily), Version 3.26. NOAA National Climatic Data Center. http://doi.org/10.7289/V5D21VHZ [Accessed Dec 2019]. |
|  | Historical humidity, gridded data aggregated to county-level. | PRISM Climate Group, Oregon State University, http://prism.oregonstate.edu, originally created 4 Feb 2004, [Accessed Dec 2019] |
| Bias-corrected and downscaled temperature and precipitation projections | Localized Constructed Analogs (LOCA) contain daily temperature (max and min) and precipitation data for a range of CMIP5 climate scenarios, baseline, and projection years. | U.S. Bureau of Reclamation, Climate Analytics Group, Climate Central, Lawrence Livermore National Laboratory, Santa Clara University, Scripps Institution of Oceanography, U.S. Army Corps of Engineers, and U.S. Geological Survey, 2016: Downscaled CMIP3 and CMIP5 Climate Projections: Release of Downscaled CMIP5 Climate Projections, Comparison with Preceding Information, and Summary of User Needs. Data available at: <http://gdo-dcp.ucllnl.org/downscaled_cmip_projections/>. |
| Time Use Data | American Time Use Survey (ATUS), multi-year microdata files. | U.S. Bureau of Labor Statistics. American Time Use Survey. Available at https://www.bls.gov/tus/datafiles-0318.htm |
|  | Additional location variables from the Current Population Survey (CPS) | Sarah Flood, Miriam King, Renae Rodgers, Steven Ruggles and J. Robert Warren. Integrated Public Use Microdata Series, Current Population Survey: Version 7.0 [dataset]. Minneapolis, MN: IPUMS, 2020. https://doi.org/10.18128/D030.V7.0 |
|  | Census delineation files provide a crosswalk between Census statistical areas and county FIPS codes. | U.S. Census Bureau. Delineation Files. https://www.census.gov/geographies/reference-files/time-series/demo/metro-micro/delineation-files.html |
| High-risk workers | Number of non-agricultural workers by industry in 2009. Used to calculate hourly wage rates with BLS weekly wage data. | U.S. Bureau of Labor Statistics. 2009. Table 21. Persons at work in nonagricultural industries by class of worker and usual full- or part-time status. Available at <https://www.bls.gov/cps/aa2009/cpsaat21.pdf>. |
|  | Number of agricultural workers in 2009. Used to calculate hourly wage rates with BLS weekly wage data. | U.S. Bureau of Labor Statistics. 2009. Table 19. Persons at work in agriculture and related and in nonagricultural industries by hours of work. Available at <https://www.bls.gov/cps/aa2009/aat19.txt>. |
|  | County-level high-risk workers, used to estimate total lost wages. | U.S. Census Bureau. American Community Survey 2008-2012. Table 12 Sex by Industry for the Civilian Employed Population 16 Years and Over.  Downloaded at county-level from: Steven Manson, Jonathan Schroeder, David Van Riper, and Steven Ruggles. IPUMS National Historical Geographic Information System: Version 14.0 [Database]. Minneapolis, MN: IPUMS. 2019. http://doi.org/10.18128/D050.V14.0 |
| Wage rates | Weekly wages by industry, 2009. Used to calculate hourly wage rate with two BLS hours-worked datasets. | U.S. Bureau of Labor Statistics. 2010. Quarterly Census of Employment and Wages. See Table 2 “Private industry by six-digit NAICS industry and government by level of government, 2009 annual averages: Establishments, employment, and wages, change from 2008”. Available at https://www.bls.gov/cew/publications/employment-and-wages-annual-averages/2009/tables/private-industry-by-six-digit-naics-and-government-by-level-of-government.pdf. |
| Population projections | Median Variant Projection of the United Nation’s (UN) 2015 *World Population Prospects* dataset used to project future U.S. population for 2015-2100. | United Nations, 2015: World Population Prospects: The 2015 Revision. United Nations, Department of Economic and Social Affairs, Population Division. Data available at: <https://population.un.org/wpp/> |
|  | U.S. national and county-level population figures from 2000-2015 | U.S. Census Bureau, cited 2017: Population Estimates Program. Available online at <https://www.census.gov/programs-surveys/popest.html> |
|  | County-scale population and developed land projections from the Integrated Climate and Land-Use Scenarios model (version 2). The spatial pattern of population change in ICLUSv2 relies on assumptions regarding fertility, migration rate, and international immigration – these were parameterized based on the Shared Socioeconomic Pathway (SSP) 2, which suggests medium levels of fertility, mortality, and international immigration. | Population projection model documentation available at this link <https://www.epa.gov/iclus>  EPA, 2017: Updates to the Demographic and Spatial Allocation Models to Produce Integrated Climate and Land Use Scenarios (ICLUS) (Version 2). U.S. Environmental Protection Agency, Washington, DC, EPA/600/R-16/366F. Available online at <https://cfpub.epa.gov/ncea/iclus/recordisplay.cfm?deid=322479>  Additional information about projection dataset can be found at:  U.S. Environmental Protection Agency. Multi-Model framework for quantitative sectoral impacts analysis: A technical report for the Fourth National Climate Assessment. Washington, DC; 2017. Available at https://cfpub.epa.gov/si/si_public_record_Report.cfm?Lab=OAP&dirEntryId=335095. |
| Domestic economic growth | Projection of future gross domestic product from the Emissions Predictions and Policy Analysis (EPPA, v6) model.  The projection of GDP growth through 2040 from the 2016 Annual Energy Outlook reference case is used to calibrate EPPA-6, and is also then combined with EPPA-6 baseline assumptions for other regions and time periods | Chen, Y.-H. H., et al. The MIT EPPA6 Model: Economic Growth, Energy Use, and Food Consumption. MIT Joint Program on the Science and Policy of Global Change, Report 278, Cambridge, MA (2015)  U.S. Energy Information Administration, 2016: Annual Energy Outlook. Available online at https://www.eia.gov/outlooks/archive/aeo16/ |
| Price deflator | Dollar years are adjusted to $2015 using the U.S. Bureau of Economic Affairs’ Implicit Price Deflators for Gross Domestic Product, Table 1.1.9. | U.S. Bureau of Economic Analysis. 2020. Table 1.1.9 Implicit Price Deflators for Gross Domestic Product. Available at <https://bea.gov/national/index.htm>. Downloaded August 31, 2020. |
