# Supplementary material for: Temperature and work: Time allocated to work under varying climate and labor market conditions
Source: PLoS One. 2021 Aug 25;16(8):e0254224. doi: 10.1371/journal.pone.0254224 (PMC8386856; doi:10.1371/journal.pone.0254224)
Supplement: S1 Text — (DOCX) [file pone.0254224.s006.docx]

**S1 Text. Climate Projection Models and Scenarios**

Climate stressor information is input into the model in the form of one historical climate dataset, six future projections, and two greenhouse gas (GHG) emissions scenarios. These scenarios are similar to those used in the preceding Climate Change Impacts and Risk Analysis (CIRA) studies. The future climate projections are a subset of those generated for the Intergovernmental Panel on Climate Change’s Fifth Assessment Report (AR5). For climate forcing, two Representative Concentration Pathways (RCPs) are used: RCP8.5 and RCP4.5. RCP8.5 represents a future with substantial warming caused by higher GHG emissions, resulting in a total change in radiative forcing of 8.5 W/m^2^ by 2100 (compared to 1750). Of the four available RCP scenarios, RCP8.5 aligns best with historical cumulative CO_2_ emissions and projections out to 2050 under current and stated policies (Schwalm et al., 2020). Research is ongoing as to the likelihood of the high levels of GHG emissions in RCP8.5 after 2050 in the absence of a global climate policy (Christensen et al., 2018; Burgess et al., 2020; Hausfather & Peters, 2020; Schwalm et al., 2020).

RCP4.5 represents a future with significant global reductions in GHG emissions, achieving a total radiative forcing of 4.5 W/m^2^ by 2100. Of the many GCMs generated for the AR5 as part of the Coupled Model Intercomparison Project Phase 5 (CMIP-5; Taylor et al. 2012), this study uses the following six: CanESM2, CCSM4, GFDL-CM3, GISS-E2-R, HadGEM2-ES, and MIROC5.

These projections were downscaled using a statistically-based process that employs a multi-scale spatial matching scheme to select analog days from observations across CONUS (Pierce et al. 2014). This dataset, LOCA (Localized Constructed Analogs; U.S. Bureau of Reclamation et al, 2016) has a spatial resolution of 1/16 degree for daily maximum temperature, daily minimum temperature, and daily precipitation.

As in most impacts work, the selection of a subset of general circulation models (GCMs) is necessary due to computational, time, and resource constraints. As such, six GCMs were chosen (summarized in Table 1) with the intent of ensuring that the subset captures a large range of the variability in climate outcomes observed across the entire ensemble from the CMIP-5.

**Table SM1-1: Summary of the six GCMs used in the analysis**

| Center (Modeling Group) | Model Acronym | References |
| --- | --- | --- |
| Canadian Centre for Climate Modeling and Analysis | CanESM2 | Von Salzen et al.  2013 |
| National Center for Atmospheric Research | CCSM4 | Gent et al. 2011;  Neale et al. 2013 |
| NASA Goddard Institute for Space Studies | GISS-E2-R | Schmidt et al. 2006 |
| NOAA Geophysical Fluid Dynamics Laboratory | GFDL-CM3 | Donner et al. 2011 |
| Met Office Hadley Centre | HadGEM2-ES | Collins et al., 2011;  Davies et al. 2005 |
| Atmosphere and Ocean Research Institute, National Institute for Environmental Studies, and Japan Agency for Marine-Earth Science and Technology | MIROC5 | Watanabe et al.  2010 |

While many different metrics could be used in this type of comparison, a logical approach is to compare the projections from CMIP5 CGMs for annual and seasonal temperature and precipitation. While these averaged metrics may not be perfect substitutes for comparing extreme weather effects, the relationship should be sufficiently strong for selecting climate models from the broader ensemble.

To aid in the selection of GCMs, the LASSO tool was used to produce scatter plots showing the variability across the CMIP5 ensemble for projected changes (2085-2095 compared to the 1986-2005 reference period) in annual and seasonal temperature and precipitation. The national-scale plots are shown in Figures SM 1-1 and SM-8. The GCMs used in the climate projections for this paper are displayed with blue circles around them to highlight their location within the scatter plots. The model identified as the double median across temperature/precipitation outcomes shown in a red rectangle.

**Figure SM 1-1. Variability of projected annual temperature and precipitation change across the CMIP5 ensemble for the continental United States**


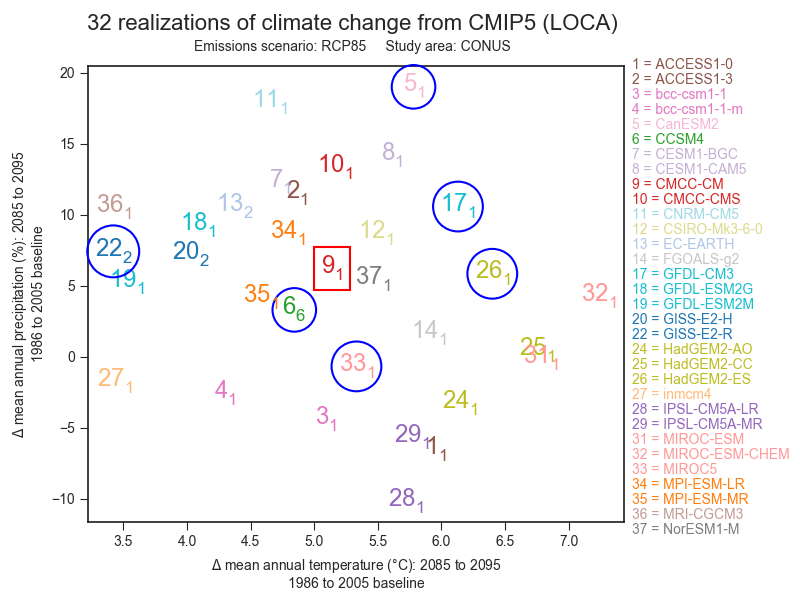


**Figure SM 1-2. Variability of projected summertime temperature and precipitation change across the CMIP5 ensemble for the continental United States**


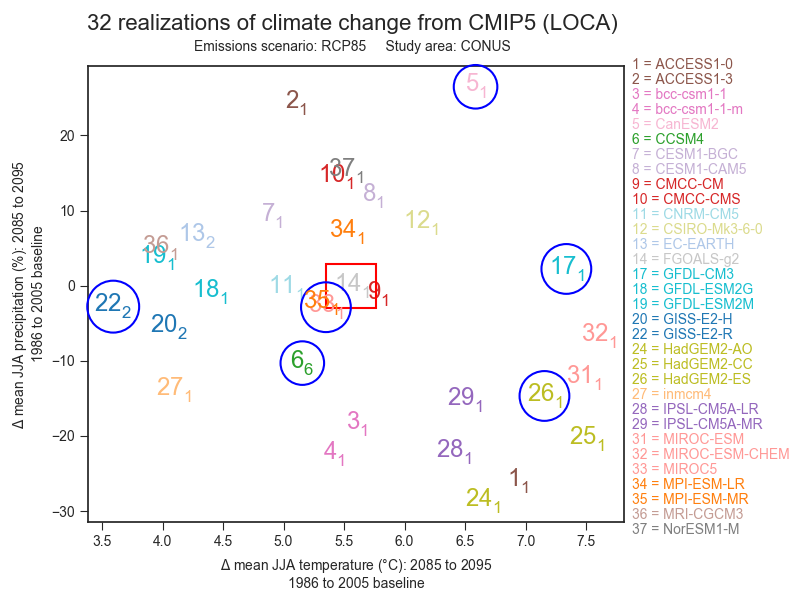


As shown in Figures SM 1-1 and 1-2, the six selected GCMs (CanESM2, CCSM4, GFDL-CM3, GISS-E2-R, HadGEM2-ES, and MIROC5) cover a large range of the variability across the entire ensemble in terms of annual and summertime temperature and precipitation. This selection also balances the range alongside considerations of model independence, broader usage by the scientific community, and skill at reproducing observed climate. Sanderson et al. (2015a, 2015b) provide analysis of both model skill at the global scale and independence of underlying code. These criteria were considered in the selection process. Number of degree days above 90 (the key climate metric used in this analysis) are shown in Figure SM-3.

**Figure SM1-3: Mean projected number of degree days above 90 degrees (°F) per year for the six climate models, two emissions scenarios, and the 2050 (2040-2069) and 2090 (2080-2099) eras.**


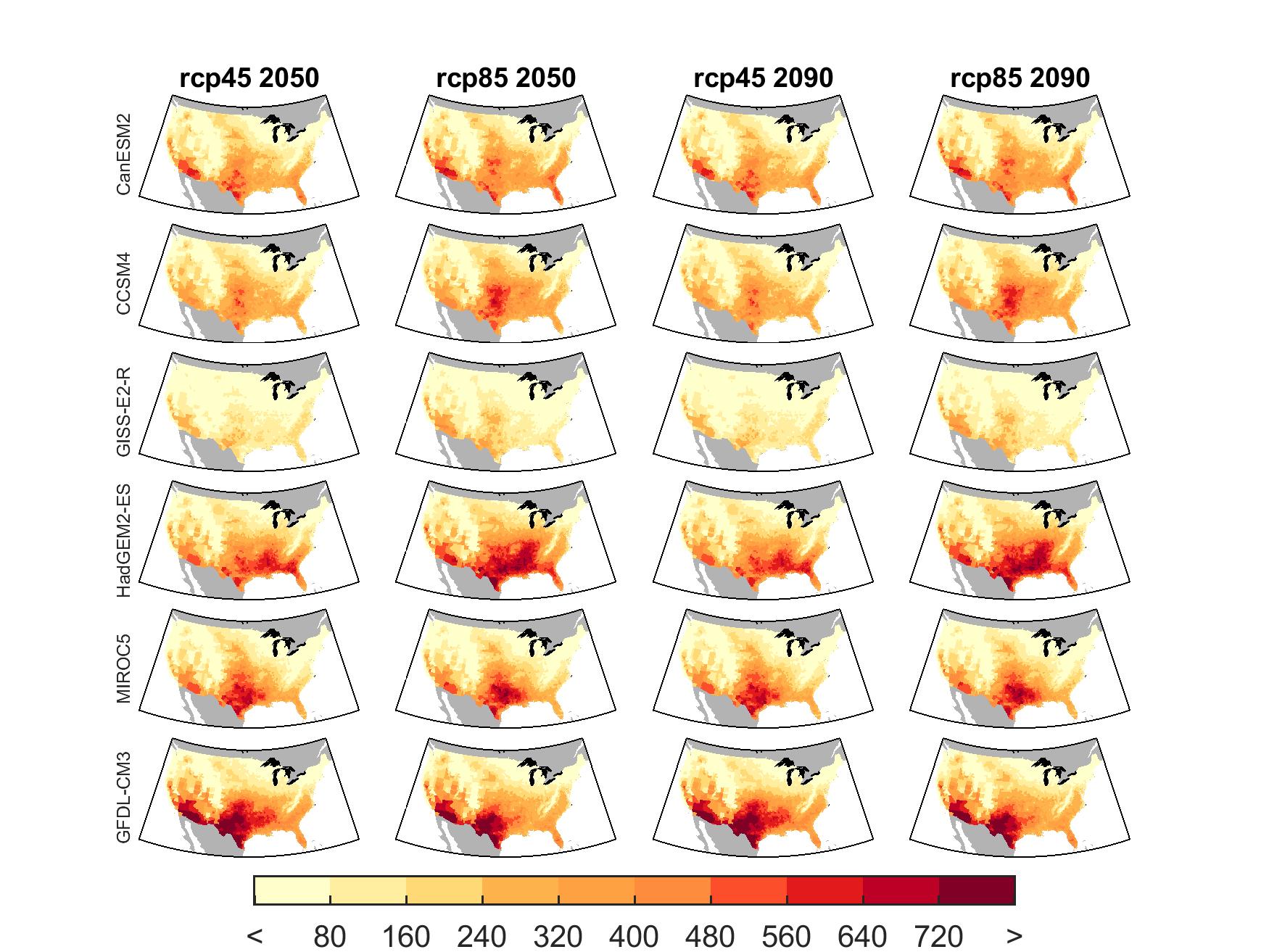


**References**

Burgess, M. G., Ritchie, J., Shapland, J., & Pielke, R., Jr. (2020). IPCC baseline scenarios over-project CO_2_ emissions and economic growth, *SocArXiv*, 18 Feb. 2020. http://doi.org/10.31235/osf.io/ahsxw

Christensen, P., Gillingham, K., & Nordhaus, W. (2018). Uncertainty in forecasts of long-run economic growth, *Proc. Natl. Acad. Sci. USA*, 115, 5409–5414, doi:10.1073/pnas.1713628115.

Collins WJ, Bellouin N, Doutriaux-Boucher M, Gedney N, Halloran P, Hinton T, Hughes J, Jones CD, Joshi M, Liddicoat S, Martin G. 2011. Development and evaluation of an Earthsystem model–HadGEM2. Geoscience Model Develeopment, 4:1051-1075.

Davies, T., Cullen, M. J. P., Malcolm, A. J., Mawson, M. H., Staniforth, A., White, A. A. & Wood, N. (2005) A new dynamical core for the Met Office’s global and regional modelling of the atmosphere. Q. J. R. Meteor. Soc. 131, 1759–1782. doi:10.1256/qj.04.101

Donner, Leo J., et al. (2011), The dynamical core, physical parameterizations, and basic simulation characteristics of the atmospheric component AM3 of the GFDL Global Coupled Model CM3. Journal of Climate, 24(13), doi:10.1175/2011JCLI3955.1.

Gent PR, Danabasoglu G, Donner LJ, Holland MM, Hunke E, Jayne S, Lawrence D, Neale RB, Rasch PJ, Vertenstein M, Worley PH. 2011. The community climate system model version 4. Journal of Climate, 24:4973-4991.

Hausfather, Z., & Peters, G. P. (2020). Emissions—the ‘business as usual’ story is misleading, *Nature* 577, 618–620.

Neale RB, Richter J, Park S, Lauritzen PH, Vavrus SJ, Rasch P, Zhang M. 2013. The mean climate of the community Atmosphere Model (CAM4) in forced SST and fully coupled experiments. Journal of Climate, 26:5150-5168.

Pierce, D.W.; Cayan, D.R.; Thrasher, B.L. Statistical downscaling using localized constructed analogs (LOCA). J Hydrometeorology, 2014, 15(6):2558-2585.

Sanderson B, Knutti R, Caldwell P (2015a) A representative democracy to reduce interdependency in a multimodel ensemble. Journal of Climate. doi: 10.1175/JCLI-D-14-00362.1

Sanderson B, Knutti R, Caldwell P (2015b) Addressing interdependency in a multi-model ensemble by interpolation of model properties. Journal of Climate. doi: 10.1175/JCLI-D-14-00361.1

Schmidt GA, Ruedy R, Hansen JE, Aleinov I, Bell N, Bauer M, Bauer S, Cairns B, Canuto V, Cheng Y, Del Genio A. 2006. Present-day atmospheric simulations using GISS ModelE: Comparison to in situ, satellite, and reanalysis data. Journal of Climate, 19:153-192.

Schwalm, C. R., Glendon, S., & Duffy, P. B. (2020). RCP8.5 tracks cumulative CO_2_ emissions, *Proc. Natl. Acad. Sci. USA*, 117, 19656–19657, doi:10.1073/pnas.2007117117.

Taylor KE, Stouffer RJ, Meehl GA (2012) An overview of CMIP5 and the experiment design. Bulletin of the American Meteorological Society. doi:10.1175/BAMS-D-11-00094.1

U.S. Bureau of Reclamation et al. Downscaled CMIP3 and CMIP5 climate projections –addendum release of downscaled CMIP5 climate projections (LOCA) and comparison with preceding information. September 2016, data available here: http://gdo-dcp.ucllnl.org/downscaled_cmip_projections/

von Salzen K, Scinocca JF, McFarlane NA, Li J, Cole JN, Plummer D, Verseghy D, Reader MC, Ma X, Lazare M, Solheim L. 2013. The Canadian fourth generation atmospheric global climate model (CanAM4). Part I: representation of physical processes. Atmosphere-Ocean, 51:104-125.

Watanabe M, Suzuki T, O'ishi R, Komuro Y, Watanabe S, Emori S, Takemura T, Chikira M, Ogura T, Sekiguchi M, Takata K. 2010. Improved climate simulation by MIROC5: mean states, variability, and climate sensitivity. Journal of Climate 23:6312-6335.
