# Supplementary material for: Temperature and work: Time allocated to work under varying climate and labor market conditions
Source: PLoS One. 2021 Aug 25;16(8):e0254224. doi: 10.1371/journal.pone.0254224 (PMC8386856; doi:10.1371/journal.pone.0254224)
Supplement: S2 Text — (DOCX) [file pone.0254224.s007.docx]

**S2 Text. Comparison to Previous Estimate (Martinich and Crimmins, 2019)**

The lost wages estimated in this study are about half those estimated in Martinich and Crimmins (2019), which were based on the damage function from Graff-Zivins Neidell (2014). Here we discuss the identifiable differences in approach and input data between the 2019 estimate and the update.

- 2019 estimate **scaled number of high-risk workers** over time in proportion to population growth; Update holds number of high-risk workers constant.
- 2019 estimate included a slightly smaller **base population of high-risk workers** than the Update.
- 2019 estimate was based on an economic growth period and did not **adjust for the likelihood of future recession periods**; Update makes and adjustment for growth/recession likelihood.
- 2019 estimate used only five GCMs; Update includes the original five GCMs and **adds GFDL** which predicts higher temperatures on average.
- 2019 estimate assumed **lower average hourly rates** than the Update.

The table below shows how each of these factors contribute to the difference in results. Note that these multipliers may not be exact as the data used for the 2014 CIRA estimates were recreated for purposes of this comparison but should give a sense of the relative impact of each difference. If we apply the multipliers for population growth and the recession period adjustment (the two most significant methods changes) to the new estimates we would get $132,910 in 2090, RCP8.5 compared to the original $155,087.

The remaining difference can be attributed to differences in the estimate. First, this update considers a longer base period (2003-2007; 2015-2018^[[1]](#footnote-1)^) than the 2019 estimate (2003-2007). Second, the estimates measure two slightly different outcomes. The 2019 model estimated additional hours lost relative to a 70-degree day, compared to baseline losses, and the current model estimates additional hours lost per additional degree day above 90 degrees, compared to baseline losses.

**Table S2-1. Comparison of results**

|  | **2090, RCP8.5** | | **2019 to Update Multiplier** |
| --- | --- | --- | --- |
|  | **2019 Estimate** | **2020 Update** |  |
| **Estimates** | | | |
| Hours Lost | 1,873 | 1,062 | **0.57** |
| Wages Lost | $155,087 | $80,012 | **0.52** |
| **Known Adjustments** |  |  |  |
| Scale HR workers with Population growth | 1.43 | 1 | 0.70 |
| Base # of HR workers | 28,926 | 33,898 | 1.17 |
| Recession/Growth Period Adj. | 1 | 0.86 | 0.86 |
| Added GCM^1^ | 1 | 1.09 | 1.09 |
| **Product of Hours Multipliers** |  |  | **0.77** |
| Base wage rate | $23.02 | $26.53 | 0.84 |
| **Product of all Multipliers** |  |  | **0.65** |

Notes:

1. Multiplier based on the 6-GCM average results versus the 5-GCM average for the 2020 Update results if we exclude GFDL.

**References:**

Graff Zivin J, Neidell M. Temperature and the allocation of time: Implications for climate change. Journal of Labor Economics. 2014 Jan 1;32(1):1-26.

Martinich J., A. Crimmins. Climate damages and adaptation potential across diverse sectors of the United States. Nature Climate Change. 2019 April 8;9(5):397-404.

1. Recession period is excluded from model that is used to forecast damages. [↑](#footnote-ref-1)
